# From COVID to fibrosis: lessons from single-cell analyses of the human lung

**DOI:** 10.1186/s40246-022-00393-0

**Published:** 2022-06-13

**Authors:** Aurelien Justet, Amy Y. Zhao, Naftali Kaminski

**Affiliations:** 1 Section of Pulmonary, Critical Care, and Sleep Medicine, Department of Internal Medicine, Yale School of Medicine, New Haven, CT USA; 2 Service de Pneumologie, Centre de Competences de Maladies Pulmonaires Rares, CHU de Caen UNICAEN, CEA, CNRS, ISTCT/CERVOxy Group, GIP CYCERON, Normandie University, 14000 Caen, France; 3 Yale University School of Medicine, New Haven, CT USA; 4 Department of Genetics, Yale University School of Medicine, New Haven, CT USA

**Keywords:** COVID-19, Fibrotic interstitial lung disease, Single-cell analysis, Idiopathic pulmonary fibrosis, IPF cell atlas

## Abstract

The increased resolution of single-cell RNA-sequencing technologies has led to major breakthroughs and improved our understanding of the normal and pathologic conditions of multiple tissues and organs. In the study of parenchymal lung disease, single-cell RNA-sequencing has better delineated known cell populations and identified novel cells and changes in cellular phenotypes and gene expression patterns associated with disease. In this review, we aim to highlight the advances and insights that have been made possible by applying these technologies to two seemingly very different lung diseases: fibrotic interstitial lung diseases, a group of relentlessly progressive lung diseases leading to pulmonary fibrosis, and COVID-19 pneumonia, an acute viral disease with life-threatening complications, including pulmonary fibrosis. We discuss changes in cell populations and gene expression, highlighting potential common features, such as alveolar cell epithelial injury and aberrant repair and monocyte-derived macrophage populations, as well as relevance and implications to mechanisms of disease and future directions.

## Introduction

Single-cell RNA-sequencing (scRNA-seq) technologies allow for the detailed analyses of the transcriptome of every single cell in each tissue or organ. This enhanced and unprecedented resolution can better classify cells and cell states as well as identify cell-specific changes in gene expression. This technology dramatically increases the detail, accuracy, and resolution of our map of the living organism [1, 2]. Indeed, in just a few years after its introduction, scRNA-seq technologies have been widely implemented, led to major breakthroughs, and improved our understanding of the normal and pathologic conditions of several tissues and organs [3]. Technological and computational breakthroughs have facilitated an exponential increase in the number of cells profiled, now having the capability to capture information from over a million cells per study [4, 5]. Thus, scRNA-seq is being used in large-scale efforts to provide a high-resolution map of every cell in the human body [6].

The lung is one of the most complex organs of the human body with over forty cell types specializing in gas exchange, surfactant production, and protection against pathogens and harmful pollution [7–9]. In recent years, advanced parenchymal lung disease has emerged as a major cause of mortality and morbidity. Chronic Obstructive Pulmonary Disease (COPD), most commonly caused by exposure to cigarette smoke but also indoor pollution [10] is now considered the 3rd leading cause of death in the world [11]. Fibrotic interstitial lung disease (fILDs), which are a heterogeneous group of lung disorders that cause progressive scarring of the lung parenchyma and are associated with substantial morbidity and mortality [12–14]. Of the fILDs, idiopathic pulmonary fibrosis (IPF) is the most lethal, with a median survival of two to five years after diagnosis. Acute respiratory distress syndrome (ARDS) is a highly lethal respiratory failure syndrome and has been under the public radar in the past, but the COVID-19 pandemic has brought it to immediate attention as it is the major cause of death in severe COVID-19. After recovering from the immediate illness, many patients have experienced long-standing symptoms, known as long-COVID [15], and it is estimated that about 15–20% of COVID-19 patients develop a transient pulmonary fibrosis-like disease one year after the infection [16], with the percentage developing long-term sequela still unknown.

**Fig. 1:**
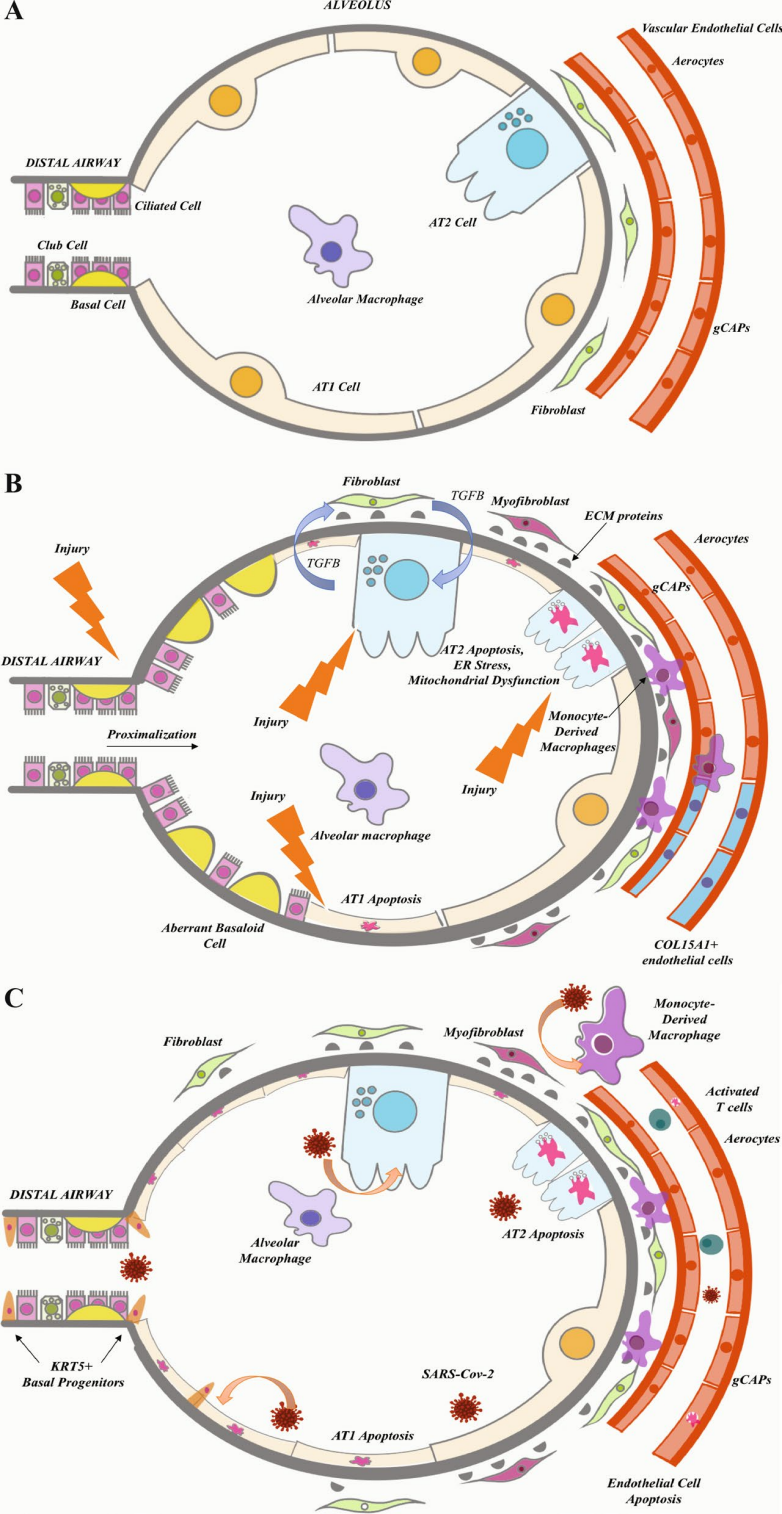
Representative figure of the distal alveolus for healthy control, IPF, and COVID-19 patient samples. Distal aberrant changes in early lungs. **A** Distal airway and alveolus from a healthy control lung. Several epithelial cell types can be found in the bronchiole epithelium, including club cell, ciliated cell, and basal cell. Basal cells are the airway epithelial progenitor. The alveoli are located in the respiratory bronchioles as scattered outpockets, extending from their lumens. Each lung contains approximately 150 million alveoli providing a surface of gas exchange of forty square meters. Each alveolus is wrapped in a fine mesh of capillaries covering about 70% of its area and composed of endothelial and venule cells. There are three major types of alveolar cells. Two types of pneumocytes or pneumonocytes are known as type 1 (AT1) and type 2 (AT2) cells that are found in the alveolar wall, and a large phagocytic cell known as an alveolar macrophage that moves about in the lumens of the alveoli and the connective tissue. AT1 cells are thin and are involved in the process of gas exchange between the alveoli and blood. AT2 cells are cuboidal and produce surfactant, a lipoprotein that reduced the alveolar tension. AT2 cells also serve as the local alveolar epithelial progenitor. The alveolar fibroblasts are located in the interstitial compartment and are the main source of ECM proteins like collagen and elastin that allow the alveoli to stretch when they fill with air during inhalation. **B** In IPF, repeated injuries of a senescent alveolar epithelium and bronchiole can lead to the loss of alveolar resident AT1 and AT2 cells, and an increase in airway epithelial cells, such as club cells, basal cells, ciliated cells suggesting “proximalization” of the distal lung. In the vascular compartment, there is a loss in alveolar capillary cells corresponding to gCAP and aerocytes with a concomitant ectopic increase in venous ECs (*COL15A1*^*pos*^) in the lung parenchyma. Immunohistochemistry (IHC) confirmed their presence in fibrotic and bronchiolized areas in IPF lungs paralleling the proximalization of the distal lung observed in the epithelium. In addition to changes in these well-described lung resident cell populations, a new subpopulation of cells distinct was identified and called aberrant basaloid cells. Regarding the immune cells, monocyte-derived macrophages also contribute to fibrosis through increased recruitment and extravasation of these cells, as well as their secreted molecules that also lead to a profibrotic environment. The change of the cellular composition of the epithelial, immune, and endothelial compartments lead to an abnormal secretion of profibrotic mediators such as TGF-*β* and MMP7 and to the differentiation of alveolar fibroblasts into myofibroblasts. These cells are postulated as the main source of aberrant production of ECM deposition in the interstitial space, ultimately leading to the destruction of the alveolar space and to fibrosis of the lung. **C** During end-stage COVID-19 lung infection, a similar but different process occurs. SARS-Cov-2 preferentially infects AT2 and AT1 cells, monocyte-derived macrophages, and endothelial cells, causing apoptosis of AT1, AT2, and endothelial cells. To compensate for ATI and ATII cell loss, KRT5 + basal progenitors proliferate and migrate into the alveolus. The injury of the alveolar epithelium also leads to the differentiation of alveolar fibroblasts into myofibroblasts. These cells may contribute to the deposition of the extracellular proteins in the interstitial space. It is unclear what happens to endothelial cell proportions, though it has been postulated that these cells decrease. Viral infection load also correlates directly with gCAPs and aerocytes proportions. There is also an increase in the recruitment and extravasation of macrophages as well as an increase in activated T cells

In this review, we describe the insights derived by applying single-cell profiling technologies to parenchymal lung disease with a specific focus on fILD and COVID-19, as those have been studied most. So far, scRNA-seq technologies have been applied to samples obtained to close to 200 patients with fILD and COVID-19 as well as healthy controls. Because of the vastness of the data, we chose to organize the discussion based on distinct cell populations, covering the discovery of novel cell populations, the description of known cell populations, and the changes in gene expression within distinct cell populations. All the methodological information of the studies reviewed in this article, including samples, technology, and main findings, is summarized in Table 1. Figure 1 summarizes the perspectives developed in this paper regarding the pathophysiology of progressive lung disease in fILDs and COVID-19.

**Table 1:** Summary of single-cell RNA-sequencing studies of healthy and fibrotic human lung tissue

Authors	Sample breakdown	Number of cells	Technology used	Validation study	Data availability
Adams et al*. *[20]	32 IPF, 18 COPD, 28 healthy controls	312,928	10X Genomics 3’ Chromium system	IHC, other datasets	GEO GSE136831, www.IPFcellatlas.com
Reyfman et al*.* [19]	8 donor lung biopsies, 8 lung explants: 4 IPF, 2 SSc-ILD, 1 polymyositis, 1 CHP	76,070	10X Genomics 3’ v2 Chromium system	ISH, IHC	nupulmonary.org/resources, www.IPFcellatlas.com
Habermann et al*. *[21]	20 PF (12 IPF, 3 CHP, 2 NSIP, 2 sarcoidosis, 1 unclassifiable ILD), 10 healthy controls	114,396	10X Genomics 3’ v2 and 5’ v1 Chromium systems	ISH	GEO GSE135893, www.IPFcellatlas.com
Melms et al*.* [33]	19 severe COVID-19, 8 healthy controls	116,000	10X Genomics 3’ v3.1 Chromium system (single nuclei)	IHC, multiplexed microscopy, imaging mass cytometry	GEO GSE171524
Travaglini et al*,* [7]	3 nonsmoker and smoker controls	~ 75,000	10X Genomics 3’ Chromium system (65,662 cells) and Smart-Seq2 (9,404 cells)	ISH, flow cytometry	https://www.synapse.org/#!Synapse:syn21041850 https://hlca.ds.czbiohub.org/ European Genome-Phenome Archive EGAS00001004344
Hasan et al*. *[46]	3 moderate COVID-19, 12 severe COVID-19, 5 decreased COVID-19, 6 healthy controls (meta-analysis)	Not reported	10X Genomics 5’ and 3’	NA	GEO GSE1618215, GSE169471, GSE145926 (lungs)
Schupp et al*.* [47]	73 healthy controls (meta-analysis)	~ 15,000 vascular cells	10X Genomics	IF, ISH, IHC	GEO GSE164829, www.lungendothelialcellatlas.com
Delorey et al*.* [35]	16 deceased COVID-19 (lung-only)	106,792	10X Genomics scRNA-Seq, snRNA-seq	RNAscope, ISH	GEO GSE171668, DUOS (raw human sequencing data): duos.broadinstitute.org
Tsukui et al. [49]	3 IPF, 2 s SSc-ILD	83,316	10X Genomics 3’ v2	ISH, IHC	GEO GSE132771, GSE147066
Bahrat et al. [36]	5 severe COVID (3 explants, 2 donor)	Not reported	10X Genomics 3’ v3	ISH	GSE158127 https://github.com/NUPulmonary/2020_Bharat. Single-cell RNA-sequencing data
Morse et al. [44]	3 healthy lungs, 3 IPF lower lobe, 3 IPF upper lobe	47,771	10X Genomics 3’ v1 or v2	IHC, IF	GEO GSE128033, IPFcellatlas.com

### Epithelial cells

Injury to alveolar epithelial cells is central to all parenchymal lung diseases. In pulmonary fibrosis, loss of the following cells occurs: alveolar type I (AT1) cells, which are flat cells that make up much of the alveolar cells and carry out gas exchange, and alveolar type II cells (AT2), which secrete surfactant, regulate alveolar fluid, and serve as local progenitor cells and has been described long ago [17, 18].

Single-cell transcriptomic analysis confirms this notion. All scRNA-seq studies of fibrotic human lungs revealed loss of alveolar resident AT1 and AT2 cells and an increase in airway epithelial cells, such as club cells, basal cells, ciliated cells [19–22], suggesting “proximalization” of the distal lung. Airway basal cells, which are epithelial progenitor cells characterized by the expression of *P63*, *KRT1*7, and *KRT5* and are capable of differentiating into any type of airway cells [23] are also increased. Airway basal cells have been previously reported to be increased in the IPF lung [24], and their presence in the lavage fluid of patients with IPF indicates the worst prognosis [25]. In addition to changes in these well-described lung resident cell populations, our group identified a subpopulation of cells distinct from any previously described in the lung. We used the term aberrant basaloid cells to describe them, because they expressed common basal cell markers, such as *P63*, *LAMB3*, and *KRT17*, but did not express *KRT5* or *KRT15.* The aberrant basaloid cells have several intriguing features. They are epithelial in nature but express mesenchymal markers, including *COL1A1, TNC, HMGA2, CDH2*, which suggests a partial epithelial–mesenchymal transition [20]. Moreover, these cells express senescence-related genes, such as *CDKN1A, CDKN2A, CCND1*, *CCND2, MDM2* as well as *GDF15*, which has recently been proposed as an epithelial cell senescence marker [26, 27]. Aberrant basaloid cells also express numerous genes previously implicated in IPF, suggesting that they may play a key role in pulmonary fibrosis. *MMP7* (matrilysin), a matrix metalloproteinase that cleaves proteoglycans, fibronectin, elastin, and casein, is highly expressed in aberrant basaloid cells. MMP7 is important in pulmonary fibrosis. Mice lacking *MMP7* are relatively protected from bleomycin-induced fibrosis [28], and increased blood levels of MMP7 have been shown to distinguish IPF patients from controls and other diseases [29] and are the most validated markers of increased mortality in IPF [30, 31]. Aberrant basaloid cells also express AVB6, an epithelial-restricted integrin that is not expressed in the healthy adult human lung and is known to drive fibrosis by activating TGF-*β* by binding its latency-associated peptide (LAP) of TGF-*β* and is an emerging therapeutic target for pulmonary fibrosis [32]. Additional evidence of the potential role of these cells in driving fibrosis is that in IPF lungs, these cells consistently localize to the epithelial layer covering myofibroblast foci [20]. The cells are found in other datasets and simultaneously described by Habermann et al. [21].

Single-cell analyses of COVID-19 lung injury were mostly limited to patients with advanced COVID-19 lung disease with samples obtained at lung transplantation or postmortem [33–36]. The papers reported similar losses of loss AT2 and AT1 cells with marked transcriptional changes in the remaining cells. AT2 exhibited decreased expression of genes encoding for surfactants, such as *SFTPC* or *SFTPA* [33–35], similar to what is seen in pulmonary fibrosis. They also exhibited high expression of genes associated with host viral response, including those for programmed cell death such as *STAT, TNFSF10*, inflammation like *IRF3*, adaptive and innate immune response such as *IFI16* or *HLA-A*. AT2 cells showed a decrease of *ETV5*, a transcription factor required to maintain AT2 identity. *CAV1*, a marker of late AT1 maturation, was also expressed at significantly lower levels in the COVID-19 lung. In addition, AT2 showed enrichment for *TNF* and *HIF1a* signaling and had an overrepresentation of 53 pathways that suggested an arrest of the cellular division and a blockade in this transitional state that explains, at least partially, the decrease of AT1 cells. Recent studies have shown that inflammation can induce a cell state that is characterized by failure to fully transition to AT1 cells; this phenomenon has been coined “damage-associated transient progenitors” (DATPs), “alveolar differentiation intermediate” (ADI), or “pre-AT1 transitional cell state” (PATS) [37–39]. This program signature (*KRT8,CLDN4,CDKN1A*) is induced during lung injury [40] and is increased in COVID-19 pneumocytes [35]. These cells are distinct from aberrant basaloid cells, previously described in the IPF lung, but aberrant basaloid cells have been reported in the lungs of two patients with end-stage COVID-19 [36]. The existence of these cells must be confirmed in a larger cohort but could suggest the development of irreversible fILD, for which transplantation is likely the only therapeutic option.

The presence of tuft cells, a rare subset of epithelial cells involved in airway inflammation and intestinal tissue regeneration implicated in lung response to viral pneumonia [41], has also been postulated to be in the lungs of patients with COVID-19 pneumonia [33]. Tuft cells express *TAS2* receptors, which have been shown to attenuate allergic airway inflammation [42], *DLKC1,* and *POU2F3* and emerge ectopically in the lung after influenza virus infection [43], where they may contribute to dysplastic remodeling. In COVID-19, Melms et al*.* identify tuft cell-like subpopulations in the upper airway, which were ectopically present in the parenchyma. Further studies are needed to confirm the existence of these cells in the COVID-19 infected lung and to elucidate their implication in lung remodeling.

### Immune cells

Single-cell studies of advanced parenchymal lung disease are dominated by changes in macrophages populations. In fILDs, Reyfman et al. described a profibrotic alveolar macrophage subtype seen in patients with IPF, systemic sclerosis-associated ILD, myositis-associated ILD, and hypersensitivity pneumonitis [19]. Clustering of lung macrophage single-cell data revealed distinct subclusters that were specific to patients with lung fibrosis. In particular, genes that were differentially expressed in monocyte-derived alveolar macrophages included *CHI3L1, MARCKS2, IL1RN, PLA2G7, MMP9,* and *SPP1.* To confirm these findings, the authors performed in situ RNA hybridization to localize these putative profibrotic macrophage subtypes using *SPP1* and *CHI3L1* and confirmed alveolar macrophage heterogeneity in patients with pulmonary fibrosis [19]. Morse et al*.* sequencing nine lung samples (three healthy controls, three IPF lower lobes, and three IPF upper lobes), reported three distinct macrophage subtypes in both normal and fibrotic lungs, including (1) monocyte markers-expressing, (2) *FABP4*^*hi*^, and (3) *SPP1*^hi^ macrophages [44]. *SPP1*^hi^ macrophages were significantly increased in the lower lobes of patients with IPF, where fibrosis was more pronounced and expressed *MERTK, LGMN,* and *SIGLEC10.* We confirmed this finding and further applied archetypal analysis to three different subtypes: classical monocytes, profibrotic IPF macrophages, and control-enriched inflammatory macrophage subtype [20]. Building on these findings of what they term “IPF-expanded macrophages,” Ayaub et al*.* conducted both CyTOF and flow cytometry analyses in addition to re-analysis of aforementioned single-cell datasets [45]. Using flow cytometry, they discovered that CD84 + CD36 + macrophages were expanded in IPF compared to control and COPD lungs and that this subpopulation aligns with previously described macrophages enriched in IPF, providing a cell surface marker protein-based validation of the scRNA-seq classification.

Within the lungs of COVID-19 patients, Bharat et al*.* analyzed lung explants from patients with end-stage COVID-19 lung disease and identified a profibrotic macrophage subpopulation expressing similar markers as those found in pulmonary fibrosis patients [36]. The profibrotic monocyte-derived alveolar macrophages expressed *SPP1, ILRN, MMP9, CHI3L1,* and *PLA2G7*. Conversely, tissue-resident macrophages were nearly depleted in COVID-19 patients as compared with donor lungs from healthy controls. There were other subpopulations of macrophages that were specifically enriched in COVID-19 patients. A subset of COVID-19 samples had a subtype of macrophages expressing osteoclast-like markers, potentially in calcified and necrotic lung tissue. Furthermore, an inflammatory macrophage subset highly expressing genes involved in lipid and iron metabolism, immune signaling, and cell motility were found in a lung explant but not postmortem biopsies of COVID-19 patients. Overall, the authors found similar profibrotic macrophages in COVID-19 as in pulmonary fibrosis patients suggesting a role for this cell population in sustaining fibrosis.

Melms et al*.* [36] described aberrant activation of myeloid cells including monocytes, monocyte-derived macrophages, and resident alveolar macrophages which were more frequent in COVID-19 lungs. monocyte-derived macrophages exhibited increased expression of *CTSB*, *CTSD*, *CTSZ*, *PSAP*) and alveolar macrophages exhibited reduced expression AXL, a regulator of efferocytosis (i.e., clearance of apoptotic cells). Rendeiro et al*.* [34] found that around 8% of macrophages were positive for S protein. These macrophages exhibited higher expression of apoptosis and inflammatory markers like cleaved *CASP3, pSTAT3, KIT,* and *IL6* but were negative for complement proteins C5b-C9. Moreover, CD14 + CD16 + CD206 + CD163 + CD123 + interstitial macrophages were increased in lungs with late COVID-19 compared to healthy lungs. Monocytes in early COVID-19 displayed the highest expression of *IL1B*, which may recruit neutrophils. Indeed, in patients with COVID-19, the authors observed increased interactions between macrophages and neutrophils as well as between neutrophils and macrophages. However, inter-macrophage interactions were decreased in COVID-19. Hasan et al*.* further substantiated this claim in their meta-analysis, finding that inflammatory monocytes and macrophages were significantly increased in severe and deceased COVID-19 patients compared to those who had milder forms of the disease [46]. While the analyses are limited by small numbers of tissues, it suggests that at least in refractory cases, monocyte-derived macrophages with features similar to “profibrotic macrophages” in pulmonary fibrosis accumulate in COVID-19 lungs.

### Endothelial cells

Dysfunctional endothelial cells have been previously linked to fibrotic lung disease; however, *in vitr*o studies have been limited by difficulty with endothelial cell cultures. Thus, endothelial cells were not previously studied in earnest in the context of fibrotic lung disease until the advent of single-cell RNA-sequencing. Recently, we created a comprehensive atlas of endothelial cells in the lung [47]. We conducted a meta-analysis of lung single-cell RNA-sequencing datasets, selecting for endothelial cells (ECs) using canonical markers, including *PECAM1, CDH5, CLDN5,* and *ERG.* Endothelial cells in the lungs were broadly categorized into two subtypes: vascular (example markers include *ENG, PCDH17, CLEC14A, BMPR2, FLT1, SLCO2A1, EPAS1, GATA2)* and lymphatic (*PROX1*, *LYVE1*, *FLT4*, and *PDPN*). Within vascular ECs, we found three main subtypes: arterial, capillary, and venous. Arterial ECs are exposed to higher pressure compared to capillary and venous ECs; as such, these cells exhibit more gap and tight junctions, extracellular matrix proteins, and protease inhibitors. These cells can be identified due to their higher expression of *EFNB2, SOX17, BMX, SEMA3G, HEY1, LTBP4, FBLN5, GJA5,* and *GJA4.* Capillary ECs facilitate gas exchange in the alveoli and include aerocytes and general capillary ECs (gCAPs) in an approximate one-to-two ratio. These terms were coined by Gillich et al. [48]. Capillary ECs were distinguished by high expression of *CA4, PRX, RGCC, SPARC,* and *SGK1.* Aerocytes express *EDNRB, TBX2, FOXP2, CLEC4E, SPON2, PRKG, CHRM2, S100A4, EDA, HPGD, SOSTDC1* and do not express Weibel–Palade body-associated genes. gCAPs can be identified by the following genes: *GPIHBP1, CD36, FCN3, BTNL9, BTNL8, CD14, IL7R, IL18R1.* Finally, of the three vascular EC subtypes, venous ECs are exposed to the lowest pressure. Thus, these cells express genes that enable immune cell extravasation, including *VCAM1, SELP, SELE,* and *ACKR1*. Venous ECs are further subdivided into pulmonary venous (*COL15A1*^*neg*^), which are found in the lung parenchyma, and systemic venous ECs (*COL15A1*^*pos*^), which are localized to the airways and visceral pleura. In IPF, we observed loss in alveolar capillary cells corresponding to gCAPs and aerocytes [20] with a concomitant increase in venous ECs (*COL15A1*^*pos*^) in the lung parenchyma where they are never found. Immunhistochemistry (IHC) confirmed their presence in fibrotic and bronchiolized areas in IPF lungs paralleling the proximalization of the distal lung observed in epithelial cell populations [20].

Lung endothelial cells in COVID-19 also demonstrated distinct cell composition and phenotypic shifts compared to those of healthy controls. Delorey et al*.* identified several EC subsets in patients with COVID-19 [35]. These subsets included lymphatic (*PROX1, MMRN1, RELN, PKHD1L1, CCL21, SEMA3D*), arterial (*VEGFA, EFNB2, DKK2, FBLN5, SERPINE2, CLDN10)*, venous (*HDAC9, CPE, IL1R1, ACKR1, PTGS1*), capillary aerocytes (*EDNRB, HPGD, CA4, PRKG1, TBX2, RCSD1, EXPH5, EDA, FOXP2, CHRM2*), two capillary subtypes (*CA4, RUNX1, PRKCB and FCN3, VWF, PTPRB, NOSTRIN*), and a mixed population (*CD14, FCN3, GPIHBP1, SOSTDC1*). Unlike Rendeiro et al*.*’s finding of a decreased proportion of endothelial cells in COVID-19 patients, the authors found that patients had significantly increased numbers of vascular and lymphatic endothelial cells (FDR < 0.001) [35]. These subtypes were also determined to have the highest significantly different gene expression profiles across all cell types in the lung, with 587 and 317 out of 1580 genes with increased expression in COVID-19 in lymphatic and vascular ECs, respectively. Though the authors do not explicitly report on changed pathways, Rendeiro et al*.* found that there was a significant enrichment in C5b-C9 complement activation and apoptosis pathways in differentially expressed genes in lung endothelial cells [34]. Higher proportions of venular and capillary endothelial ECs were also directly correlated with higher viral burden, possibly suggesting the role of these subtypes in disease [35]. Thus, endothelial cell changes in COVID-19 still present a muddled picture and require further investigation. Based on reported findings thus far, unlike changes in epithelial and myeloid cells, there was a limited similarity between fILD and COVID-19 lungs, potentially reflecting the distinct impact of viral pathogenesis on the endothelial compartment.

### Mesenchymal compartment

Pulmonary fibrosis is characterized by aberrant activation of fibroblast cells with accumulation of cells defined as myofibroblasts, changes in lung resident fibroblast populations, and excessive deposition of extracellular matrix proteins. In the last decade, a lot of studies investigated the molecular and metabolic changes involved in this differentiation. However, little is known about the diversity of fibroblast cellular populations in the IPF lung, mainly due to the lack of specific markers for the distinct populations. Single-cell analysis provides a more comprehensive view of cellular identity, where global transcriptomic features, rather than singular cell markers, are exploited. Single-cell analysis confirmed the relative increase of fibroblasts in general and of myofibroblasts in the pulmonary fibrosis patients compared to controls [20, 21]. These cells showed enrichment of fibrillar collagen contents of *ACTA2* in the fibrotic lung versus normal lung. Despite a lot of effort dedicated to the investigation of the cellular source of myofibroblast, the origin of this cell is still debated. Habermann et al*.* identified four distinct lung fibroblast phenotypes expressing pathologic ECM proteins and localized in a different area of the fibrotic lung [21]. Myofibroblasts were predominantly localized in subepithelial regions around airways and areas of cystic remodeling, while HAS1^hi^ fibroblasts appear restricted to the immediate subpleural region; PLIN2 + and other LUM + fibroblasts are found diffusely in parenchymal regions. The distinct phenotypes are probably not stable over time *in vivo*. Indeed, lung fibroblast populations demonstrate considerable plasticity. Application of lineage reconstruction technique partition-based graph abstraction suggested that pathologic, *ACTA2*-expressing IPF myofibroblasts are connected to a quiescent stromal population also present in control lungs [20]. Recent scRNA-seq data showing the higher *ACTA2* expression in smooth muscle cells and pericytes suggest that α-SMA may not be a specific marker of pathologic fibroblasts producing the highest levels of extracellular matrix protein [49]. Using several independent datasets, Tsukui et al*.* [49] identified a unique population of cells that expressed the highest levels components of the pathologic extracellular matrix protein and were marked by high levels of expression of *CTHRC1* in the human and mice fibrotic lung. The highest *CTHRC1* RNA and protein expression was observed within fibroblastic foci, which are known as the hallmark lesion characterizing human pulmonary fibrosis [50]. Two different computational analyses (RNA velocity and pseudotime trajectory) suggested that these cells most likely differentiated from a population of alveolar fibroblasts. Considering the critically important role of fibroblast populations in fibrosis, and the progress in understanding other cell types, there is a need for a more focused analysis of mesenchymal cell populations in human pulmonary fibrosis.

In COVID-19 lungs, several single-cell studies confirmed the significant increase of the fibroblast and myofibroblast population, compared to control lung [33–35]. A positive correlation between the degree of fibrosis, which was determined by Red Sirius staining reflecting collagen level, and the disease duration. In the same study, the authors identified four different clusters of fibroblasts identified as adventitial, alveolar, intermediate pathologic, and pathologic. These last two categories were significantly increased in the COVID-19 lung compared to the control lung. These cells expressed *CTHRC1, COL1A1, COl3A1*. A ligand–receptor analysis, realized across all cell types, revealed that the enriched inferred ligand–receptor interactions were *TGFB1-TGFBR2* and *BMP6-ACVR1* which belong to the TGF-β family and superfamily, respectively. TGF-β is the key mediator involved in the fibroblast-to-myofibroblast differentiation but also in the pre-alveolar type 1 transitional cell state [33, 35]. Like in pulmonary fibrosis, despite the potentially important role of fibroblasts in COVID-19 lung disease, the information is relatively limited.

### Challenges and perspectives

COVID-19-induced lung disease and fILD represent distinct and very different disease entities. fILD represent a group of chronic parenchymal lung diseases with irreversible and progressive lung scarring characterized in its most lethal form by a distinct histopathological pattern of usual interstitial pneumonia in which fibroblastic foci represent the leading edge of fibrotic destruction of the lung, whereas severe COVID-19 induced fibrosis, is acute, post-viral, occurs in a subset of the patients and seems to be related to the severity of the infection and some comorbidities [51]. The application of scRNA-seq to those two different conditions allowed identification that aberrant activation of repair pathways and a relative change in the distribution of some cell populations were similar. Both sets of conditions exhibited a relative loss of alveolar epithelial cells populations associated with a relative increase of airway epithelial cells. In both diseases, the decrease of alveolar epithelial cells seemed to be the consequence of an increase in cell death pathways and potentially a stunted, or failed, compensatory regeneration response. Aberrant basaloid cells, population that is never seen in the healthy lung, but emerges in the lung parenchyma of patients with pulmonary fibrosis were also seen COVID-19 induced lung fibrosis. The cellular source of aberrant basaloid cells is still debated [20, 21] but their emergence in conditions associated with end-stage fibrosis suggests that they have a significant role in the process, as is also supported by their gene expression profiles. Regarding immune cells in the lung, a profibrotic monocyte-derived alveolar macrophage has been extensively described in both patients with pulmonary fibrosis and end-stage COVID-19. This finding suggests that there may be an aberrant immune process driving profibrotic macrophage proliferation that feeds into fibroblast and myofibroblast activation that contribute to fibrosis. Endothelial cell populations also undergo shifts in COVID-19 and pulmonary fibrosis, but these do not seem to overlap. In particular, patients with IPF have a marked decrease in capillary ECs (both aerocytes and general capillary ECs) and an increase in systemic venous ECs that highly express *COL15A1.* COVID-19 patient lungs have been found to have increased vascular and lymphatic endothelial cells, with aerocyte and capillary EC involvement directly correlating with disease burden, and evidence for an endothelial inflammatory response. This may represent a substantial difference, as endothelial injury seems to represent an integral and critically important in COVD-19 lung injury [33, 35] but in fILD, the endothelial changes seem to be secondary to the primary disease process. All of the above findings are summarized in Fig. 1.

scRNA-seq is revolutionizing our perceptions of cellular phenotypes. First, a cell type was defined by its shape and location; identification of cell surface markers used for flow cytometry and immunochemistry led to a more complex classification of immune cell types populations; ascertainment of cell-specific transcription factors, cytoskeletal proteins, and secreted molecules improved the classification of lung resident cells; and staining for cytokines, their receptors, and cell cycle markers allowed statements with relation to activation states of these cells. However, these markers, while frequently validated, only represent a small fraction of the proteome, are heavily driven by observations in mice, and do not capture the complexity and granularity of cell changes. Thus, it should not come as a surprise that the application of scRNA-seq to human tissues generated such a wealth of novel information. What was unexpected was the reproducibility of the data, with similar features emerging from multiple independent datasets as well the emergence of common features of lung injury and response including the disruption of the alveolar niche, the loss of specialized alveolar epithelial cells, the emergence of aberrant cellular populations, including aberrant basaloid cells and profibrotic macrophages, which was a phenomenon common to fILD and COVID-19 infected lungs. In fact, it may be argued that the creation of single-cell transcriptomic atlases may have a bigger impact than large genome-wide association studies on the campaign to cure these diseases [22] especially when other fILD such as those with autoimmune etiologies are studied in greater depth [52].

When assessing this impact of scRNA-seq we should also be cognizant of the technical, biological, and computational limitations of the technology [53]. The most frequent technical challenge is related to the batch effect, which refers to changes in gene expression that are due to experimental or technical factors. Transient biological states can mask the underlying cell identity and constitute a biological challenge. For example, cell cycle phases can confound cell-type identity in differentiating cells. The inherently noisy and sparse nature of scRNA-seq makes it difficult to impute what is a true biological “zero” versus a technical dropout; thus, a large computational challenge that remains in the field is denoising and imputing the data. Another challenge for reproducing scRNA-seq findings across different studies is clustering. While it is supposed to be unsupervised, most clustering methods include one or more parameters that can be chosen by the user. These choices can have a large effect on the outcomes of the clustering. Consequently, the most important step of this kind of work is the validation of the computational analysis at the protein or mRNA level. In this review, we highlighted findings that were consistent across several studies and were also supported by validation methods such as immunostaining or in situ hybridization (Table 1), thus highly likely to be reproducible. However, this may suggest that more changes were overlooked. An additional limitation is the lack of spatial insight. While scRNA-seq analysis identifies cell subpopulations within a tissue, it does not capture their spatial distribution nor reveal local networks of intercellular communication acting in situ [54]. In the lung, the tightly regulated three-dimensional organization of the distinct alveolar cell populations is critically important for its’ function, and potentially the loss of this organization, critical to understanding disease pathogenesis. Methods that allow cell resolution spatial transcriptomic profiling, like MERFISH [55], GeoMx Digital Spatial Profiling [33], or Dbit-Seq [56] would provide an important additional layer to understand the cellular interaction networks that govern lung remodeling and scarring in response to injury.

## Conclusions

In conclusion, the application of scRNA-seq to parenchymal lung diseases such as fILD and severe COVID-19 pneumonia allowed a detailed characterization of cellular subpopulations and phenotypes, with the unexpected discovery of similar responses among epithelial and myeloid cells. Despite technological limitations, these findings are reproducible and suggest key roles for profibrotic monocyte-derived macrophages and aberrant basaloid cells in lung response to injury and scarring that could have potential therapeutic implications. Analysis of additional samples potentially at earlier stages of disease and with a focus on other cellular populations and the addition of spatial high throughput cellular and molecular profiling technologies will greatly enhance the value of these observations.

## Abbreviations

scRNA-seq: Single-cell RNA-sequencing
fILD: Fibrotic interstitial lung disease
IPF: Idiopathic pulmonary fibrosis
SSc-ILD: Scleroderma-associated ILD
CHP: Chronic hypersensitivity pneumonitis
COPD: Chronic obstructive pulmonary disease
ARDS: Acute respiratory distress syndrome
IHC: Immunohistochemistry
IF: Immunofluorescence
ISH: In situ hybridization
gCAP: General capillary endothelial cell
EC: Endothelial cell
AT1: Alveolar type 1 epithelial cell
AT2: Alveolar type 2 epithelial cell
DATP: Damage-associated transient progenitors
ADI: Alveolar differentiation intermediate
PATS: Pre-AT1 transitional cell state
LAP: Latency-associated peptide

## References

[1] Börner K, Teichmann SA, Quardokus EM, Gee JC, Browne K, Osumi-Sutherland D (2021). Anatomical structures, cell types and biomarkers of the human reference atlas. Nat Cell Biol.

[2] Osumi-Sutherland D, Xu C, Keays M, Levine AP, Kharchenko PV, Regev A (2021). Cell type ontologies of the human cell atlas. Nat Cell Biol.

[3] Karlsson M, Zhang C, Méar L, Zhong W, Digre A, Katona B, Sjöstedt E, Butler L, Odeberg J, Dusart P, Edfors F, Oksvold P, von Feilitzen K, Zwahlen M, Arif M, Altay O, Li X, Ozcan M, Mardinoglu A, Fagerberg L, Mulder J, Luo Y, Ponten F, Uhlén M, Lindskog C (2021). A single–cell type transcriptomics map of human tissues. Sci Adv.

[4] Hwang B, Lee JH, Bang D (2018). Single-cell RNA sequencing technologies and bioinformatics pipelines. Exp Mol Med.

[5] Kharchenko PV (2021). The triumphs and limitations of computational methods for scRNA-seq. Nat Methods.

[6] Svensson V, Vento-Tormo R, Teichmann SA (2018). Exponential scaling of single-cell RNA-seq in the past decade. Nat Protoc.

[7] Travaglini KJ, Nabhan AN, Penland L, Sinha R, Gillich A, Sit RV (2020). A molecular cell atlas of the human lung from single-cell RNA sequencing. Nature.

[8] Franks TJ, Colby TV, Travis WD, Tuder RM, Reynolds HY, Brody AR (2008). Resident cellular components of the human lung: current knowledge and goals for research on cell phenotyping and function. Proc Am Thorac Soc.

[9] Schiller HB, Montoro DT, Simon LM, Rawlins EL, Meyer KB, Strunz M (2019). The human lung cell atlas: a high-resolution reference map of the human lung in health and disease. Am J Respir Cell Mol Biol.

[10] Hansel NN, McCormack MC, Belli AJ, Matsui EC, Peng RD, Aloe C (2013). In-home air pollution is linked to respiratory morbidity in former smokers with chronic obstructive pulmonary disease. Am J Respir Crit Care Med.

[11] WHO Global Health Estimates [Internet]. Available from: https://www.who.int/news-room/fact-sheets/detail/the-top-10-causes-of-death

[12] Travis WD, Costabel U, Hansell DM, King TE, Lynch DA, Nicholson AG (2013). An official American thoracic society/European respiratory society statement: update of the international multidisciplinary classification of the idiopathic interstitial pneumonias. Am J Respir Crit Care Med.

[13] Olson AL, Gifford AH, Inase N, Fernández Pérez ER, Suda T (2018). The epidemiology of idiopathic pulmonary fibrosis and interstitial lung diseases at risk of a progressive-fibrosing phenotype. Eur Respir Rev.

[14] Selman M, Pardo A (2021). When things go wrong: exploring possible mechanisms driving the progressive fibrosis phenotype in interstitial lung diseases. Eur Respir J.

[15] Nalbandian A, Sehgal K, Gupta A, Madhavan MV, McGroder C, Stevens JS (2021). Post-acute COVID-19 syndrome. Nat Med.

[16] Wu X, Liu X, Zhou Y, Yu H, Li R, Zhan Q (2021). 3-month, 6-month, 9-month, and 12-month respiratory outcomes in patients following COVID-19-related hospitalisation: a prospective study. Lancet Respir Med.

[17] King TE, Schwarz MI, Brown K, Tooze JA, Colby TV, Waldron JA (2001). Idiopathic pulmonary fibrosis: relationship between histopathologic features and mortality. Am J Respir Crit Care Med.

[18] Selman M, Pardo A (2006). Role of epithelial cells in idiopathic pulmonary fibrosis: from innocent targets to serial killers. Proc Am Thorac Soc.

[19] Reyfman PA, Walter JM, Joshi N, Anekalla KR, McQuattie-Pimentel AC, Chiu S (2019). Single-cell transcriptomic analysis of human lung provides insights into the pathobiology of pulmonary fibrosis. Am J Respir Crit Care Med.

[20] Adams TS, Schupp JC, Poli S, Ayaub EA, Neumark N, Ahangari F, Chu SG, Raby BA, DeIuliis G, Januszyk M, Duan Q, Arnett HA, Siddiqui A, Washko GR, Homer R, Yan X, Rosas IO, Kaminski N (2020). Single-cell RNA-seq reveals ectopic and aberrant lung-resident cell populations in idiopathic pulmonary fibrosis. Sci Adv.

[21] Habermann AC, Gutierrez AJ, Bui LT, Yahn SL, Winters NI, Calvi CL, Peter L, Chung M-I, Taylor CJ, Jetter C, Raju L, Roberson J, Ding G, Wood L, Sucre JMS, Richmond BW, Serezani AP, McDonnell WJ, Mallal SB, Bacchetta MJ, Loyd JE, Shaver CM, Ware LB, Bremner R, Walia R, Blackwell TS, Banovich NE, Kropski JA (2020). Single-cell RNA sequencing reveals profibrotic roles of distinct epithelial and mesenchymal lineages in pulmonary fibrosis. Sci Adv.

[22] Neumark N, Cosme C, Rose K-A, Kaminski N (2020). The idiopathic pulmonary fibrosis cell atlas. Am J Physiol-Lung Cell Mol Physiol.

[23] Rock JR, Barkauskas CE, Cronce MJ, Xue Y, Harris JR, Liang J (2011). Multiple stromal populations contribute to pulmonary fibrosis without evidence for epithelial to mesenchymal transition. Proc Natl Acad Sci USA.

[24] Smirnova NF, Schamberger AC, Nayakanti S, Hatz R, Behr J, Eickelberg O (2016). Detection and quantification of epithelial progenitor cell populations in human healthy and IPF lungs. Respir Res.

[25] Prasse A, Binder H, Schupp JC, Kayser G, Bargagli E, Jaeger B, Hess M, Rittinghausen S, Vuga L, Lynn H, Violette S, Jung B, Quast K, Vanaudenaerde B, Yan X, Hohlfeld JM, Krug N, Herazo-Maya JD, Rottoli P, Wuyts WA, Kaminski N (2019). BAL cell gene expression is indicative of outcome and airway basal cell involvement in idiopathic pulmonary fibrosis. Am J Respir Crit Care Med.

[26] Zhang Y, Jiang M, Nouraie M, Roth MG, Tabib T, Winters S (2019). GDF15 is an epithelial-derived biomarker of idiopathic pulmonary fibrosis. Am J Physiol Lung Cell Mol Physiol.

[27] Kage H, Borok Z (2012). EMT and interstitial lung disease: a mysterious relationship. Curr Opin Pulm Med.

[28] Zuo F, Kaminski N, Eugui E, Allard J, Yakhini Z, Ben-Dor A (2002). Gene expression analysis reveals matrilysin as a key regulator of pulmonary fibrosis in mice and humans. Proc Natl Acad Sci U S A.

[29] Rosas IO, Richards TJ, Konishi K, Zhang Y, Gibson K, Lokshin AE (2008). MMP1 and MMP7 as potential peripheral blood biomarkers in idiopathic pulmonary fibrosis. PLoS Med.

[30] Richards TJ, Kaminski N, Baribaud F, Flavin S, Brodmerkel C, Horowitz D (2012). Peripheral blood proteins predict mortality in idiopathic pulmonary fibrosis. Am J Respir Crit Care Med.

[31] Clynick B, Corte TJ, Jo HE, Stewart I, Glaspole IN, Grainge C (2021). Biomarker signatures for progressive idiopathic pulmonary fibrosis. Eur Respir J.

[32] Sheppard D (2015). Epithelial–mesenchymal interactions in fibrosis and repair. Transforming Growth factor-β activation by epithelial cells and fibroblasts. Ann Am Thorac Soc.

[33] Melms JC, Biermann J, Huang H, Wang Y, Nair A, Tagore S (2021). A molecular single-cell lung atlas of lethal COVID-19. Nature.

[34] Rendeiro AF, Ravichandran H, Bram Y, Chandar V, Kim J, Meydan C (2021). The spatial landscape of lung pathology during COVID-19 progression. Nature.

[35] Delorey TM, Ziegler CGK, Heimberg G, Normand R, Yang Y, Segerstolpe Å (2021). COVID-19 tissue atlases reveal SARS-CoV-2 pathology and cellular targets. Nature.

[36] Bharat A, Querrey M, Markov NS, Kim S, Kurihara C, Garza-Castillon R, Manerikar A, Shilatifard A, Tomic R, Politanska Y, Abdala-Valencia H, Yeldandi AV, Lomasney JW, Misharin AV, Scott Budinger GR (2020). Lung transplantation for patients with severe COVID-19. Sci Transl Med.

[37] Kobayashi Y, Tata A, Konkimalla A, Katsura H, Lee RF, Ou J (2020). Persistence of a regeneration-associated, transitional alveolar epithelial cell state in pulmonary fibrosis. Nat Cell Biol.

[38] Strunz M, Simon LM, Ansari M, Kathiriya JJ, Angelidis I, Mayr CH (2020). Alveolar regeneration through a Krt8+ transitional stem cell state that persists in human lung fibrosis. Nat Commun.

[39] Choi J, Park J-E, Tsagkogeorga G, Yanagita M, Koo B-K, Han N (2020). Inflammatory signals induce AT2 cell-derived damage-associated transient progenitors that mediate alveolar regeneration. Cell Stem Cell.

[40] Ting C, Aspal M, Vaishampayan N, Huang SK, Riemondy K, Wang F, et al. Fatal COVID-19 ARDS associated with incomplete AEC1 differentiation from the transitional state without senescence or fibrosis. bioRxiv. 2021;2021.01.12.426404.

[41] Westphalen CB, Asfaha S, Hayakawa Y, Takemoto Y, Lukin DJ, Nuber AH (2014). Long-lived intestinal tuft cells serve as colon cancer-initiating cells. J Clin Invest.

[42] Sharma P, Yi R, Nayak AP, Wang N, Tang F, Knight MJ (2017). Bitter taste receptor agonists mitigate features of allergic asthma in mice. Sci Rep.

[43] Rane CK, Jackson SR, Pastore CF, Zhao G, Weiner AI, Patel NN (2019). Development of solitary chemosensory cells in the distal lung after severe influenza injury. Am J Physiol Lung Cell Mol Physiol.

[44] Morse C, Tabib T, Sembrat J, Buschur KL, Bittar HT, Valenzi E (2019). Proliferating SPP1/MERTK-expressing macrophages in idiopathic pulmonary fibrosis. Eur Respir J.

[45] Ayaub E, Poli S, Ng J, Adams T, Schupp J, Quesada-Arias L (2021). Single cell RNA-seq and mass cytometry reveals a novel and a targetable population of macrophages in idiopathic pulmonary fibrosis. Cell Biol.

[46] Hasan MZ, Islam S, Matsumoto K, Kawai T (2021). Meta-analysis of single-cell RNA-seq data reveals phenotypic switching of immune cells in severe COVID-19 patients. Comput Biol Med.

[47] Schupp JC, Adams TS, Cosme C, Raredon MSB, Yuan Y, Omote N, Poli S, Chioccioli M, Rose KA, Manning EP, Sauler M, DeIuliis G, Ahangari F, Neumark N, Habermann AC, Gutierrez AJ, Bui LT, Lafyatis R, Pierce RW, Meyer KB, Nawijn MC, Teichmann SA, Banovich NE, Kropski JA, Niklason LE, Pe'er D, Yan X, Homer RJ, Rosas IO, Kaminski N (2021). Integrated single-cell atlas of endothelial cells of the human lung. Circulation..

[48] Gillich A, Zhang F, Farmer CG, Travaglini KJ, Tan SY, Gu M (2020). Capillary cell-type specialization in the alveolus. Nature.

[49] Tsukui T, Sun K-H, Wetter JB, Wilson-Kanamori JR, Hazelwood LA, Henderson NC (2020). Collagen-producing lung cell atlas identifies multiple subsets with distinct localization and relevance to fibrosis. Nat Commun.

[50] Jones MG, Fabre A, Schneider P, Cinetto F, Sgalla G, Mavrogordato M (2016). Three-dimensional characterization of fibroblast foci in idiopathic pulmonary fibrosis. JCI Insight.

[51] Hu B, Guo H, Zhou P, Shi Z-L (2021). Characteristics of SARS-CoV-2 and COVID-19. Nat Rev Microbiol.

[52] Cottin V, Lega J-C, Coury F, Nasser M (2022). A call for evidence in connective tissue diseases-associated interstitial lung disease. Joint Bone Spine.

[53] Kiselev VY, Andrews TS, Hemberg M (2019). Challenges in unsupervised clustering of single-cell RNA-seq data. Nat Rev Genet.

[54] Marx V (2021). Method of the year: spatially resolved transcriptomics. Nat Methods.

[55] Chen KH, Boettiger AN, Moffitt JR, Wang S, Zhuang X (2015). Spatially resolved, highly multiplexed RNA profiling in single cells. Science.

[56] Liu Y, Yang M, Deng Y, Su G, Enninful A, Guo CC (2020). High-spatial-resolution multi-omics sequencing via deterministic barcoding in tissue. Cell.

